# Defibrillator Lead Perforation Leading to Concerning Electrocardiogram Findings: Case Report

**DOI:** 10.5811/cpcem.1466

**Published:** 2024-03-25

**Authors:** Bryan Rosenberg, Max Hockstein, Cyrus Hadadi

**Affiliations:** * Medstar Washington Hospital Center; Department of Emergency Medicine; Washington, District of Columbia; † Medstar Washington Hospital Center; Department of Cardiology; Washington, District of Columbia

**Keywords:** *case report*, *ICD*, *lead perforation*, *current of injury*, *ECG*

## Abstract

**Introduction:**

Implantable cardioverter-defibrillator (ICD) lead perforation through the myocardium may result in chest pain and electrocardiogram (ECG) changes concerning for ST-segment elevation myocardial infarction. The clinical context of the ECG aids in appropriate management.

**Case Report:**

We report the case of a 71-year-old woman experiencing chest pain after an ICD placement two weeks earlier. On presentation, she exhibited ST-segment elevation on her ECG. Computed tomography confirmed ICD lead migration. The patient’s hemodynamics were normal, and she was discharged home after a five-day hospital stay following a lead revision.

**Conclusion:**

Although rare, ICD lead perforation is a potential cause of chest pain and ischemic ECG changes. Emergency physicians should consider lead perforation as a potential differential diagnosis when evaluating chest pain in patients with ICDs, taking into account the potential complications of coronary angiography.

Population Health Research Capsule What do we already know about this clinical entity?

*Lead perforation of an implantable cardioverter-defibrillator (ICD) is a rare (0.14%) but potentially life-threatening complication of ICD placement.*

What makes this presentation of diseasse reportable?

*Our case appears to be the first presentation of chest pain and new ST-segment elevations on electrocardiogram (ECG) from right ventricular lead perforation.*

What is the major learning point?

*History of recent ICD placement with ST-segment elevation should prompt concern for lead migration. Anticoagulating a patient with a perforated ICD can lead to mortality.*

How might this improve emergency medicine practice?

*Emergency physicians should be aware of this rare but important addition to their differential diagnosis when contextualizing a worrisome ECG with a clinical history.*

## INTRODUCTION

We present a case of a right ventricular (RV) implantable cardioverter-defibrillator (ICD) lead perforating the pericardium, resulting in a patient experiencing chest pain with an electrocardiogram (ECG) concerning for ST-segment elevation myocardial infarction (STEMI). While rare, RV lead migration is crucial for emergency medicine physicians to identify. Our case appears to be the first presentation of chest pain and new ST-segment elevation from RV lead perforation. The consequence of misclassifying this presentation may have grave consequences.

## CASE REPORT

**Image 1.**
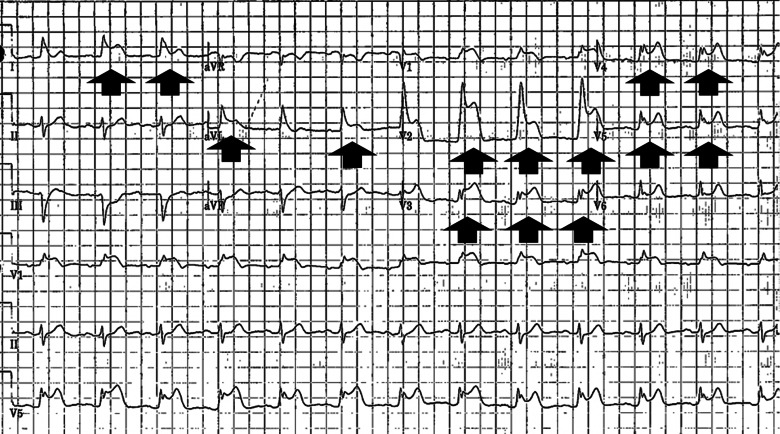
Initial electrocardiogram on presentation to the emergency department showing ST-segment elevations (arrows) across the precordial and high lateral leads.

A 71-year-old female presented to the emergency department (ED) with mild pleuritic chest pain and a twitching felt in her left chest. The patient had recently incurred a cardiac arrest due to ventricular fibrillation and had undergone ICD placement for secondary prevention two weeks earlier. She was seen earlier in the day at electrophysiology clinic and was noted to have changes in RV lead impedances and diaphragmatic stimulation during threshold testing. Therefore, she was sent to the ED for further management. Upon arrival to the ED, an ECG was performed (Image 1), which showed a normal sinus rhythm, left axis deviation, and significant ST-segment elevation across the precordial and high lateral leads.

**Image 2.**
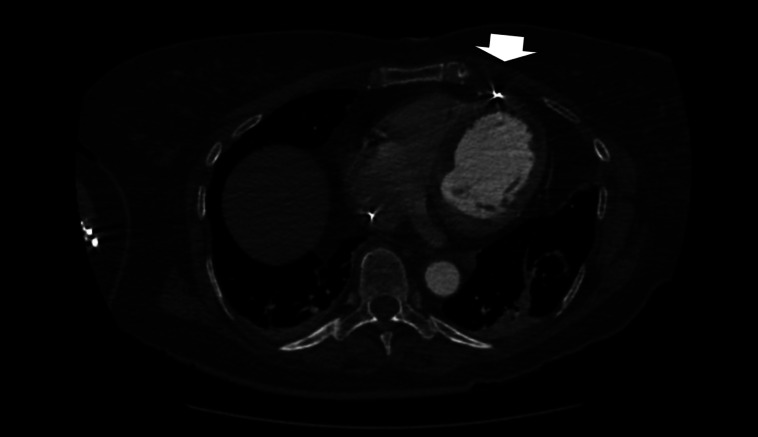
Computed tomography chest showing the right ventricular lead tip perforating into the pericardiac fat (arrow).

With a history of ischemic-type chest pain, this ECG was concerning for an acute STEMI. However, with the patient’s history of recent ICD placement in addition to large and diffuse ST-segment elevations, it was deemed more likely to be an ICD complication causing a current of injury than an ischemic event. A high sensitivity troponin was found to be within normal limits, and point-of-care ultrasound showed preserved left ventricular function and no evidence of pericardial effusion. Computed tomography chest demonstrated the RV lead tip extending through the myocardium, epicardial fat, and pericardium, with its tip in the pericardiac fat, again without evidence of pericardial effusion or mediastinal collection (Image 2).

**Image 3.**
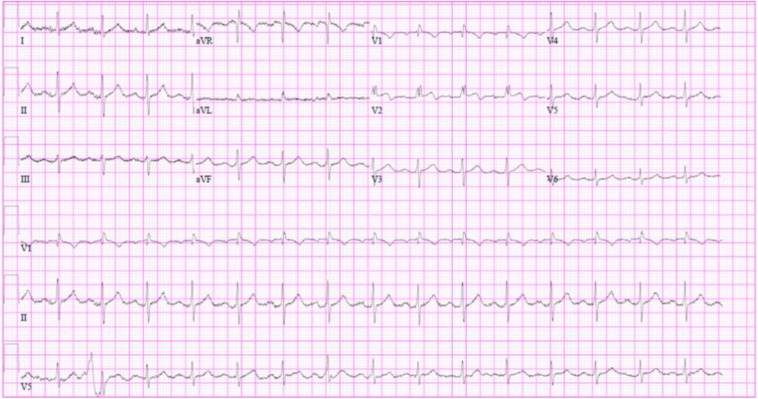
Electrocardiogram showing resolution of ST-segment elevations after perforated pacer lead was successfully revised.

The patient exhibited no hemodynamic perturbations throughout her ED stay, and she was admitted to the electrophysiology service for continued care. During her hospital stay, serial troponins remained low, and the perforated RV lead was revised. The ECG from after the RV lead revision showed resolution of the ST-segment changes (Image 3). Her hospital course was complicated by an episode of ventricular tachycardia with an effective ICD shock in the setting of likely coronary vasospasm. The patient recovered well and was discharged five days after initial presentation.

## DISCUSSION

A combination of chest pain with concerning ECG findings may prompt immediate interventional cardiology consultation for STEMI in the correct clinical context. In this case, the added information from the patient’s electrophysiology visit earlier that day provided the necessary context to guide care. Changes in RV lead impedance and diaphragmatic stimulation during threshold testing provided the clinicians with a more likely explanation for the patient’s twitching sensation and ECG changes.

Implantable cardioverter-defibrillator lead perforation is a rare (0.14%) but potentially life-threatening complication of ICD placement.^1^ Predictors for perforation of ICD include older age, female gender, left bundle branch block, worsened heart failure class, higher than normal left ventricular ejection fraction, and non–single-chamber ICD implant.^1^ The majority of pacemaker or defibrillator lead perforations are either acute or sub-acute, occurring within 30 days of implantation. Delayed or chronic perforations occurring after 30 days of implantation are uncommon. There are isolated reports of ICD leads perforating anatomic structures including ribs without pleural or pericardial effusion or other complications, the mediastinum complicated by sepsis, and the coronary sinus ostium presenting without symptoms.^2^^–^^5^

In this presentation, there was no arrhythmia, pericardial effusion, hematoma, or other dangerous pathology found. It appears to be a novel presentation of an ECG concerning for STEMI leading to a diagnosis of RV lead migration. The pathophysiology of ECG changes from pacer lead perforation relates to current of injury, which can be described as the flow of current from the damaged area of the heart to the undamaged portion. The damaged area remains depolarized, and the current of injury points toward the undamaged area, leading to the ST-segment elevations seen on our patient’s ECG.

Any patient with an ICD, chest pain, and ECG changes requires immediate intervention if hemodynamically unstable. If vital signs are normal, workup should include serial troponins and ECGs. If there is concern for lead perforation, advanced cardiac imaging with or without electrophysiology consultation should be considered.

## CONCLUSION

Implantable cardioverter-defibrillator lead perforation is a rare complication of lead implantation as evidenced by the minimal available literature surrounding its epidemiology and clinical course. The constellation of chest pain and ischemic ECG changes in patients with ICDs is even more rare. Not only does this case broaden the required differential of ECGs demonstrating current of injury to include ICD lead perforation, but it also serves as guidance for the emergency physician tasked with working up chest pain and ECG changes in a patient with an ICD. Clinicians should likewise appreciate the potential for misdiagnosis, leading to potential complications such as pericardial effusion associated with anticoagulation in a catheterization laboratory. The appreciation of device lead complications should provide the emergency physician with a less common but important addition to their differential diagnosis when contextualizing a worrisome ECG with a clinical history.

While there is no clear guidance that can be provided to physicians given the limited experience, there are three take-home points we believe every emergency physician should keep in mind when evaluating a patient with an ICD and chest pain:

1. History of recent ICD placement with ST-segment elevation should prompt concern for lead migration: without an electrophysiology visit earlier that day cluing the authors into potential lead perforation, interventional cardiology would have likely been called for possible intervention. Imaging is required to secure the diagnosis.
2. Anticoagulating a patient with a perforated ICD can lead to mortality: should the patient’s history be unrecognized, the patient would be at risk for receiving anticoagulation during coronary angiography, potentially leading to life-threatening pericardial tamponade.
3. Predictors for perforation of ICD include older age, female gender, left bundle branch block, worsened heart failure class, higher left ventricular ejection fraction, and non-single-chamber ICD implant^1^: these factors, in combination with the ECG and full clinical picture, may help guide the emergency and cardiology teams when presented with a patient with ICD placement and ECG changes, especially within 30 days of ICD implantation.
